# Prevalence of Trichomonas vaginalis infection in Kashan city, Iran (2012-2013)

**Published:** 2014-07

**Authors:** Mohsen Arbabi, Zohreh Fakhrieh, Mahdi Delavari, Amir Abdoli

**Affiliations:** *Department of Medical Parasitology and Mycology, School of Medicine, Kashan University of Medical Sciences, Kashan, Iran. *

**Keywords:** *Trichomonas vaginalis*, *Vaginal discharge*, *Culture*, *Prevalence*, *Iran*

## Abstract

**Background:** Trichomonas vaginalis infection is one of the most common sexually transmitted diseases in humans. T.vaginalis is a parasitic protozoan with a predilection for human urogenital tract and causative agent for vaginitis, cervicitis and urethritis in females. T.vaginalis infection is associated with risk of Human Immunodeficiency Virus infectivity and pregnancy complication.

**Objective:** In this study, the prevalence of T.vaginalis in individuals who referred to public health units in Kashan city, Iran was investigated.

**Materials and Methods: **This study was conducted on 970 women and 235 men who referred to 5 government health centers in Kashan, Iran during October 2012 to August 2013. Demographic information was collected as per the study protocol. Vaginal discharges and urine samples were obtained and examined by Trypticase-Yeast Extract Maltose (TYM) culture medium and wet-mount methods. The prevalence of T. vaginalis was determined using culture based method and wet-mount examinations.

**Results: **The overall prevalence of trichomonal infection was 2% (95% CI, 2±0.08). The age of infected individual was 33.7±9.4 years. All of those infected, were married housewives and 58.3% of them had primary school education. No statistical correlation was observed between clinical manifestations and parasitological results (p=0.8).

Conclusion: This study showed a relatively low prevalence of T.vaginalis infection in the study population. Since the clinical signs of trichomoniasis are the same of other Sexually Transmitted Diseases (STDs), confirmatory laboratory tests are necessary. Due to adverse outcomes of disease, there is a great need for public education regarding implementation of personal hygienic measures and prevention of inappropriate sexual contacts.

## Introduction

Trichomoniasis, is a common cause of vaginitis. It is a nonulcerative sexually transmitted disease, and is caused by the single-celled protozoan parasite named *Trichomonas vaginalis* producing mechanical stress on host cells of the human urogenital tract human (1, 2). In 2010 it was repeated that prevalence of *T.vaginalis *was 2.7% and, 1.4% in women and men, respectively (3). Trichomoniasis has been got a lot of attention as a major public health problem in recent years due to the association between *T.vaginalis* infection and human immunodeficiency virus acquisition in both men and women. The relation between *T.va*ginalis infection and human immunodeficiency virus (HIV) infection is bidirectional, such that T.vaginalis infection increases risk of transmission of HIV, and HIV infection increases transmission to *T.vaginalis* infection (4). 

In addition, this pathogen has been associated with serious health consequences including may cause a woman to deliver a low-birth-weight or premature infant, and increase chances of cervical cancer (5). Clinical manifestations differ between men and women. Infected women may be asymptomatic or have various symptoms, including a yellowish-green frothy discharge, pruritis, dysuria, and the “strawberry cervix” which is characterized by punctuates hemorrhagic lesions. In men, infection is generally asymptomatic, and can be characterized as carriers for parasite (6). 

Prevalence estimates vary between populations, but it has common range from 5-74% in women and 5-29% in men (2, 3). Detection of *T.vaginalis* due to limited factors includes: low sensitive screening techniques and nonspecific symptoms have emerged. Recently, diagnostic advances have improved our knowledge about regional and national epidemiology of trichomoniasis (7). *T.vaginalis* detection based on sensitive diagnostic tools has improved with the advent of more sensitive diagnostic techniques, in particular polymerase chain reaction (PCR). In order to accurately diagnose, at least two techniques, such as wet mount microscopy and culture have a better chance of detection the parasite. Diagnosis of trichomoniasis by PCR was found to be highly specific and sensitive, but its cost effectiveness limits its use in routine diagnostic laboratories (7, 8). 

In Iran, various studies have determined the prevalence of trichomoniasis between 2% to 8% that according to the cultural and social status can also reach over 30% (9-10). Because in recent years, trichomoniasis has emerged as one of the most common STDs and limited data are available on the epidemiology in local and national levels, this study was to determine the prevalence of *T.vaginalis* infection in females and males referred to government health centers in Kashan, Iran. 

## Materials and methods

This cross sectional study was carried out on 1205 of men and women who referred to 5 government health centers in Kashan City, from October 2012 to August 2013. All of the sexually active females and males (16-60 years old), non-pregnant females, and HIV negative individuals were examined for *T.vaginalis *infection by wet mount and culture. The referred women who had used vaginal agents and, consumption of antibiotic during the past two weeks were excluded from the study. 

After obtaining informed consent from all referred individuals, demographic information such as age, education, occupation, number of sexual partners, and clinical signs and symptoms of the genital tract were collected through interview and clinical examination by one gynecologist. Sampling was performed by expert midwife in two sterile swabs from urine, vagina wall and dorsal fornix. 

The first swab was put to glass slide with a drop of Ringer serum for microscopic examination, and the second was transported of the TYM culture medium. The cultures were transported to the parasitology laboratory, and incubated at 37^o^C for 72 hours. All samples were observed microscopically for the presence of *T.vaginalis* daily for 7 days. The motile trichomonas were reported as positive, and no parasite specimens were considered as negative for *T.vaginalis*. The research was carried out according to the local ethics review committee of Kashan University of Medical Sciences which approved the study protocols.

**Statistical analysis**

The statistical package “SPSS software (Statistical Package for the Social Sciences, version 16.0, SPSS Inc, Chicago, Illinois, USA)” was used for analysis. To compare the prevalence *T.vaginalis* between groups, Chi square analysis was employed. Significance was set at a p-value lower than 0.05. 

## Results

**Figure 1:**
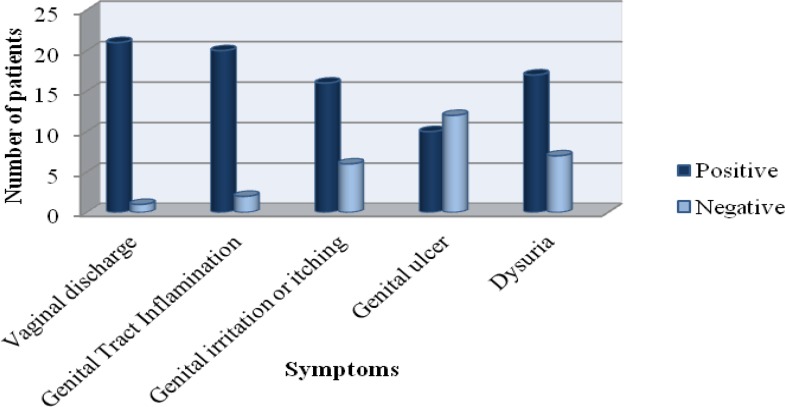
Distribution of clinical symptoms of *Trichomoniasis* among 1205 women and men

*T.vaginalis* was detected in 24 out of 1205 participants (2%) (95% CI, 1.92-2.08%) (Table I). The infected individual’s age range was 15 to 60 years old (33.7±9.4). The highest infection rate (1.4%) in women was in the age group 25-34 years, that was statistically significant compared to other age groups (p<0.05). Regarding literacy, more of the infected individuals (58.3%) had primary school education (p<0.05).Those with less education were two times more likely to have trichomonal infection compared to those who completed high school. All of the infected individuals were married housewives and had unique sexual partner. From 1205 studied persons, 24 male and female were infected, whereas 15 of them were confirmed by clinical examination. There was not statistically correlation between clinical and parasitological diagnosis method. Also, there were no significant differences between culture and mount methods for detection of *T. vaginalis.* The most predominant signs and symptoms were vaginal discharge, and the genital tract inflammation (Figure 1). There were no significant differences between this pathogen and other studied variables.

**Table I:** Comparing parasitological methods for detection of *T.vaginalis*

**Method**	**Specimen**	**Positive n (%)**	**Negative n (%)**	**Total n (%)**
Culture	Vaginal discharge	22 (2.3)	948 (97.7)	970 (100)
Wet mount	Vaginal discharge	19 (1.97)	951 (98.03)	970 (100)
Wet mount	Urine analysis (women)	17 (1.8)	953 (98.2)	970 (100)
Wet mount	Urine analysis (men)	2 (0.9)	233 (99.1)	235 (100)
Culture	women and men	24 (2)	181 (98)	1205 (100)

N: Number subject study

## Discussion

Trichomoniasis is the worldwide STDs with 248 million new cases (2). The incidence of trichomoniasis has noticeably risen especially in developing countries and in populations with high-risk behaviors such as poor sexual activity hygiene and multiple sexual partners. In Islamic countries, the prevalence of *T.vaginalis *infection was reported from 1.2% in Libya, in Jordan 3.2%, in Saudi Arabia 28.1% and India 2.1% (11-13). Poverty, socioeconomic status, low educational level, high risk sexual behaviors, and HIV positive are risk factors for acquiring vaginal trichomoniasis (2, 4, 6, 7). 

Like other parts of the world, the reports of trichomoniasis in Iran are different. The prevalence of trichomoniasis in some areas of Iran is consist of: 9.2% in Tabriz, 3.2% in Tehran, 2% in Yazd and 1.2% in Hamedan (10, 14-16). In the present study, trichomoniasis was found in 2% of female and male. The difference in the results of prevalence infection may be depended the selection of population groups, methods examination and the site of specimen collection. The present study showed, prevalence of trichomoniasis in women was higher than men. Overall symptomatic trichomoniasis is more common in women than in men. Biological differences between two sexes explain why women have a higher incidence of infection compared with men (17). 

Sex hormone is a major factor that affects epidemiology of *T.vaginalis.* Hormonal effects are likely to influence the observed differences in *T.vaginalis* incidence and prevalence between both sexes. Direct and indirect hormonal effects on the female genital tract provide a likely explanation for the greater burden of persistent infection among women compared with men. In women, hormones are important during reproductive age, directly influencing *T.vaginalis* susceptibility. Also during the reproductive years, availability of iron and oestrogen may facilitate persistent infection among females. 

Availability of iron in the female genital tract due to menstrual bleeding may contribute to sex-dependent epidemiological patterns of infection. *T.vaginalis* well adapted to existing in the setting of cyclic variation in iron availability, such as that present during menstrual cycles. As well as, the absence of oestrogen and the iron-depleted environment of the male genital tract may make men poor long-term *T.vaginalis* reservoirs. It has been hypothesised that the iron-rich environment of the vagina women provides conditions conducive to* T.vaginalis* growth and persistence. Iron facilitates the adherence of *T.vaginalis* to the genital tract epithelium, thus facilitate survival of the parasite in iron-rich environments. 

By contrast, the zinc-rich environment of the prostate remains persistent *T.vaginalis* infection in men. It is also possible that urination helps to delete *T.vaginalis* parasites from the male genital tract, whereas this mechanism would not be expected to influence clearance of vaginal secretions (18). The present study showed that even though most of infected cases were symptomatic, but reported symptoms were not specific for disease, emphasizing that clinical findings are not reliable for diagnostic purposes. 

Symptomatic trichomoniasis is more common in women than in men. *T.vaginalis *infection in women can frequently be asymptomatic carriers. However, in men tends to be less clinically apparent. Diagnosis of trichomoniasis based on only clinical symptoms should not be done due to the two reasons. First, clinical symptoms of trichomoniasis may be similar to those of other STDs. Second, clinical symptoms such as strawberry cervix and spumy discharge are seen in 2% and 12% of *T. vaginalis* infected patients, respectively (19). Some studies showed that diagnosis of trichomoniasis based only on clinical manifestations for several reasons, include the clinical symptoms may be similar with those of other STDs, the “strawberry” cervix symptom is seen in approximately 2% of patients, and frothy discharge is seen only in 12% of infected women. 

According to some studies, diagnosis of trichomoniasis using on the clinical examinations has 88% false negative and 29% false positive results. In women, over 80% of *T.vaginalis* infections are asymptomatic, and these infections can persist for several months. Symptomatic *T.vaginalis* infection in men is typically cleared spontaneously within 10 days. By contrast, symptomatic *T.vaginalis* infection in women can persist for years (20). Thus, identification of silent carriers is very important for accelerating treatment and for reducing the spread of the disease in control strategies (6). Present study showed a high prevalence of infection in sexually activity age. Similar to other STDs, trichomoniasis generally becomes more common with age, lifetime and number of sexual partners. 

Research done among various age groups women shows that a prevalence of 2.3% among adolescents aged, 4% among adults 25 years and older and 3.1% in females aged 14-49 years (3, 6, 7). In conclusion, we showed a relatively low percentage of trichomoniasis in men and women. Due to adverse outcomes of disease, at least two techniques, such as culture and PCR could be used for better diagnosis of infection. Also a range of control strategies that include education implementation of personal hygienic measures, confirmatory laboratory diagnosis, and prevention of inappropriate sexual contacts women could contribute to the disruption of transmission.
